# Advanced Thinking: Potable Reuse Strategies Gain Traction

**DOI:** 10.1289/ehp.122-A332

**Published:** 2014-12-01

**Authors:** Richard Dahl

**Affiliations:** Richard Dahl is a freelance writer in Boston. He also writes periodically for the Massachusetts Institute of Technology.

In 2011 city officials watched anxiously as the water supply for Wichita Falls, Texas, began to disappear. It was near the beginning of a historically severe drought that afflicts Texas and much of the Southwest to this day. For decades, Wichita Falls had drawn nearly all its drinking water from two reservoir lakes—Lake Arrowhead and Lake Kickapoo—but now water levels in those bodies had dropped from 88% of capacity to 55%.^1^

**Figure:**
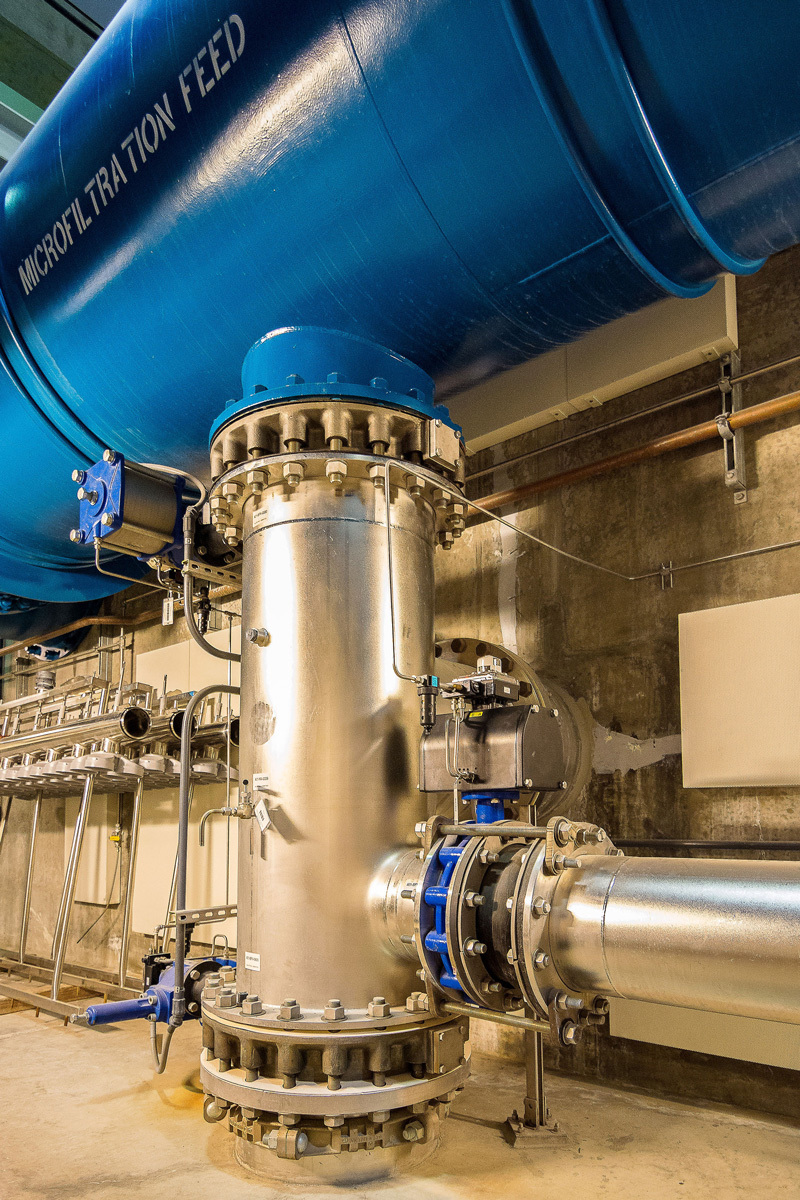
Advanced treatment capabilities enable cities to augment drinking water supplies with purified wastewater. This image shows treated water feeding into a microfiltration unit, part of the Orange County Groundwater Replenishment System. © 2014 Jon Zich/CostaMesaPhotography.com

The Wichita Falls city leaders enacted the first of what would become five increasingly tough mandatory restrictions on residential water use. But as the lakes’ shorelines continued to recede, they realized the time had come to take bolder action. “We approached the TCEQ [Texas Commission on Environmental Quality], and we said, ‘If this continues, our projections indicate that we could be out of water by 2014,’” recalls Daniel Nix, operations manager for the Wichita Falls Public Works Department.

They needed a new water source, and they had one in mind: municipal wastewater. Following an earlier drought, the city had begun supplementing its drinking water supply with poorer-quality, high-saline water from two other lakes, Kemp and Diversion, which was treated using advanced treatment technologies including microfiltration and reverse osmosis.^2^ Nix was confident the same technologies could be used to produce potable water from wastewater.

There was just one problem: neither Texas nor the federal government had any regulations governing such direct conversion of wastewater into drinking water—a process known as direct potable reuse (DPR). However, given the situation in Wichita Falls—a serious emergency coupled with readily available technology with the potential to deal with it—TCEQ said yes to trying DPR.^3^ “We sat down and worked with TCEQ to develop guidelines … that set the levels of treatment to ensure this water was completely safe for human consumption,” Nix says.

When that was done, Wichita Falls spent $13 million to build a 12-mile pipeline connecting the wastewater treatment plant to the drinking water treatment facility.^4^ The treated effluent would be further purified using advanced treatment, then blended with reservoir water at a 50/50 ratio and treated once again. Then it would be stored for approximately 24 hours in a ground storage tank before being distributed to the public.

The system went into operation on 9 July 2014, pumping million of gallons of blended water per day.^5^ “The vast majority of the people I’ve heard from say the water tastes great, that it’s actually better than the one-hundred-percent lake water,” Nix says. “The quality’s good, nobody’s gotten sick, and we haven’t had any problem with the plants. I’m very pleased with how Wichita Falls has put this together, and I think we’re showing that this can be done in the United States.”

## Widespread Need

Wichita Falls is by no means alone in scrambling to find new ways to provide potable water to citizens. In a 2011 report for the Stockholm Environment Institute, authors Frank Ackerman and Elizabeth A. Stanton concluded that the U.S. Southwest “is relying on the unsustainable withdrawal of groundwater reserves to meet today’s demand; those reserves will be drained over the next century as population and incomes grow.”^6^

One of the latest measures of the strain between water supply and human need was a 2014 study that analyzed satellite images of the Colorado River (the primary water source for much of the Southwest) taken from December 2004 to November 2013. The images showed the river lost nearly 53 million acre feet over that period, most of it as a result of groundwater depletion—water was being pumped out to replenish surface supplies.^7^

As the squeeze between supply and demand tightens, governments are taking a new look at wastewater as a valuable resource. North America generates an estimated 22 trillion gallons of wastewater annually.^8^ Three-quarters of that amount is treated to acceptable discharge standards, but only 3.8% of treated wastewater is intentionally and beneficially reused (e.g., for irrigation and industrial cooling).^8^

The reuse of wastewater for potable purposes is not a new idea. The world’s first DPR facility has provided drinking water in Windhoek, Namibia, since 1968.^9^ In 1978 the northern Virginia agency now known as Upper Occoquan Service Authority launched one of the country’s earliest indirect potable reuse (IPR) schemes, returning reclaimed water to a reservoir.^10^

In contrast to DPR, which introduces purified wastewater directly into the drinking water supply, IPR puts treated wastewater into surface waters; from there, the water percolates down to aquifers, from which it is eventually drawn for potable use. In 2007 California’s Orange County Water District (OCWD) introduced the world’s largest IPR operation, a project known as the Groundwater Replenishment System that produces 70 million gallons of highly treated wastewater per day.^11^

Mehul Patel, program manager for the Groundwater Replenishment System, says purified wastewater provides the drinking water needs of about one-fifth of the district’s 2.5 million residents. There have been no ill effects or complaints, he says, and construction is currently under way to expand output to 100 million gallons per day by May 2015.

OCWD relies on water from its large aquifer and the Santa Ana River, but cities in the southern part of Orange County depend mostly on water that’s imported from northern California and the Colorado River. “We saw this trend of heavy reliance on imported water, local river supplies not increasing, and these drought cycles coupled with increasing population,” Patel says. “So we decided early on that we should think about other alternatives and started looking at recycled or reused water as one of the potential water sources that we could use.”

The utility already had decades of experience using reverse osmosis to treat wastewater, which was then pumped underground near the coast to prevent saltwater intrusion (overuse of groundwater in coastal communities can allow saline water to push into freshwater aquifers). The reverse-osmosis facility, which had been online since 1977, was nearing the end of its life. “So we thought: what if we made it larger and used it as another source for potentially replenishing the aquifer and not just protecting the aquifer from seawater intrusion? That’s how we came up with this Groundwater Replenishment System,” Patel says.

## Regulatory Picture

Until recently, California had no regulations governing IPR,^12^ although California utilities had known for decades that the natural filtration of surface water as it percolates downward results in improved quality of the groundwater, according to Mark LeChevallier, director of innovation and environmental stewardship at American Water, a national water and wastewater utility. In the 1960s, he says, some California water systems began augmenting their aquifers by diverting surface water into holding basins.

Since purified wastewater that is now being piped to holding basins may be cleaner than surface water or even groundwater, LeChevallier suggests the primary benefit of IPR may be more psychological than actual. “They call it ‘the kiss of nature,’” he says. “You put water into a reservoir, it goes down into the ground, it mixes, and people don’t see the line of sight between treated wastewater and their drinking water.”

So if treated wastewater can be purified to superior quality levels, why not introduce it into the water supply in much faster and direct fashion, as in Wichita Falls?

**Figure:**
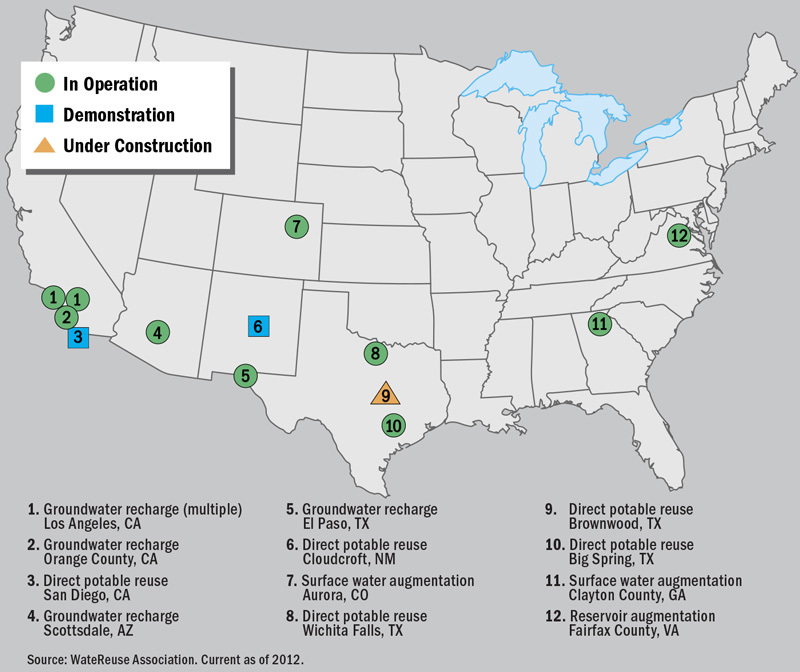
U.S. Potable Reuse Projects 1. Groundwater recharge (multiple) Los Angeles, CA 2. Groundwater recharge Orange County, CA 3. Direct potable reuse San Diego, CA 4. Groundwater recharge Scottsdale, AZ 5. Groundwater recharge El Paso, TX 6. Direct potable reuse Cloudcroft, NM 7. Surface water augmentation Aurora, CO 8. Direct potable reuse Wichita Falls, TX 9. Direct potable reuse Brownwood, TX 10. Direct potable reuse Big Spring, TX 11. Surface water augmentation Clayton County, GA 12. Reservoir augmentation Fairfax County, VA Source: WateReuse Association. Current as of 2012.

In California, at least, one answer is that “the practice generally has been deemed unacceptable in the past by regulatory agencies in the state due to a lack of definitive information related to public health protection,” as one 2010 white paper put it.^13^ Momentum and need are building, however, to allow cities and water utilities to build DPR systems. In 2009 the California State Water Resources Control Board adopted a resolution that called for an independent panel of experts to research the issue and come up with a proposal by the end of 2016 on what regulatory criteria might be put in place for that purpose.^14^

Melissa Meeker, executive director of the WateReuse Association, says that organization’s Research Foundation has allocated some $12 million for 26 research projects examining regulatory, utility, and community concerns. “The technology and safety have been well proven in indirect potable reuse schemes; this research has looked at issues such as real-time monitoring, risk assessment, and public education,” Meeker says. “But when we go to direct potable reuse, we sort of take out that environmental buffer, so you need to have higher assurances that if there’s an issue the system corrects itself instantly and doesn’t deliver tainted water.”

At the U.S. Environmental Protection Agency (EPA), regulations for drinking water are guided solely by the Safe Drinking Water Act and do not specifically address the use of treated wastewater as a drinking water source, says Peter C. Grevatt, director of the EPA Office of Ground Water and Drinking Water. But he confirms that the agency is keeping a close eye on state developments in the area of potable reuse of wastewater, in large part through discussions with state drinking water administrators.

Grevatt says the EPA is not currently considering development of any potable reuse regulations and sees its role in this area as one of supporting states that might be looking at developing regulations of their own.

## State of the Art: A Three-Step Procedure

“With indirect potable reuse, where you use the environmental buffer, we have turned a corner,” says Jeff Mosher, executive director of the National Water Research Institute, which administers the expert panel. “It used to be pretty provocative to propose that we use wastewater to augment our water supplies, but that is changing.” He predicts that most of the interest in DPR systems will come from smaller communities where reservoirs or groundwater systems are meager or lacking and where direct reuse will loom as the only viable option.

The technology that produces purified recycled wastewater has evolved over the last several decades and features multiple safeguards and redundancies, according to plant operators in California and Texas.

The first step in the purification process, microfiltration, removes solids, particulates, and bacteria at minuscule levels—OCWD uses a membrane that screens down to 0.2 µm, or about 1/300th the diameter of a human hair. The second step is reverse osmosis, which removes all organics and inorganics. The third step, known as advanced oxidation, involves a chemical reaction between hydrogen peroxide and ultraviolet light. The resulting hydroxyl radicals break down low-molecular-weight organics. Finally, minerals are put back into the water to correct its pH and hardness before it’s shipped out—whether to a holding pond or reservoir for IPR or to a tank for DPR.^9^

Although the environmental buffering involved in IPR may not necessarily make water any cleaner, Mosher points out the percolation process provides reaction time if something is found to have gone wrong during treatment. “If you put [treated wastewater] into the ground and you find out something went wrong, you have six months to do something about it; you can treat it when you pull it up,” he says. “But with direct potable, we’ve shortened that time. So what we’re studying now is how to do that monitoring even better so that we have more information to ensure the safety of the water.”

Water treatment operators say that stringent monitoring is essential to any wastewater purification operation. “A project like ours requires a lot more monitoring and online instrumentation than most regular drinking-water plants,” says OCWD’s Patel. LeChevallier says the biggest challenge facing scientists in this area is a lack of standards for chemical and microbial risk in DPR systems—and that’s where most of the research on DPR is occurring now.

“It’s clear that wastewater can be treated to a very high quality, so I wouldn’t say there are risks that we’re concerned about with projects like we see in Orange County,” Grevatt says. “But I think that with any water treatment activity, the safety of the finished drinking water is going to depend very much on the capacity of the staff and the water treatment plant to manage the quality of the source water. It’s very important to make sure, as communities are pursuing direct potable reuse projects, that the drinking water utilities have adequate funding and technical capability to manage the treatment systems that are put in place.”

## Selling Potable Reuse to the Public

Communities considering the introduction of wastewater conversion projects also need to consider the challenge of winning public support. “In people’s minds there’s a health risk if you tell them that they’re going to be drinking water that comes from sewage,” says Bahman Sheikh, a San Francisco–based water recycling consultant and distinguished fellow at the University of California, Santa Cruz, Center for Integrated Water Research.

In San Diego, for instance, efforts in 1998 to implement potable reuse met with overwhelming opposition.^15^ When San Diego residents were surveyed in 2004, they opposed potable reuse by a margin of 2 to 1.^16^ But by 2014, 79% of respondents supported a diversification strategy for drinking water that included recycling,^17^ and on 18 November 2014 the San Diego City Council unanimously approved moving forward with a full-scale IPR project.^18^ “In the intervening years, there has been considerable public education and outreach by the water agencies and local environmental groups,” says Zachary Dorsey, publications and communications manager for WateReuse. “In addition, drought conditions have worsened in California.”

The public relations successes in Orange County, San Diego, and elsewhere have provided lessons for others to follow. For instance, Meeker says, terminology makes a difference. “People are much more comfortable when you talk about reclaimed water if you call it ‘purified’ water,” she says. This isn’t just spin, either. “In many cases this is the safest drinking water available because of the high-tech treatment and the requirements for more frequent monitoring and testing,” she says.

Meeker also notes the importance of targeting messages to the populations that tend to be wariest of the idea of consuming purified wastewater—in her experience, that means mothers and women in their child-bearing years. And she says medical doctors are the most effective spokespeople for recycling wastewater.

In Wichita Falls, the Department of Public Works took local doctors and professors of environmental science and chemistry on a tour of the facilities, showing them the processes involved in the DPR system. “They were extremely comfortable with our concept,” Nix says. The city of Wichita Falls created a video^19^ about the project in which the medical and academic experts explain why the public can trust the technology.

The department also enlisted the help of the local media. “We kept them informed of every step we were taking with the TCEQ,” Nix says. “The key was keeping the public informed and not creating an information/knowledge vacuum that they would fill on their own if we didn’t fill it with correct information for them.”

Patel says a big part of selling potable reuse is simply educating people about where their drinking water comes from. “Much of the general public doesn’t realize that water has been imported for years and doesn’t naturally occur where we live,” he says. “Before you say, ‘We’re going to take this wastewater and turn it into drinking water,’ the need has to be shown. You need to lay the groundwork about why it’s necessary and economical, and then get into the science.”

As potable reuse plans gain acceptance and possibly proliferate, the challenge of gaining public support may become easier. “It’s a huge resource,” says Sheikh. “When you consider that California only recycles about eleven percent of its wastewater, the other eighty-nine percent is going into the ocean. If you think of it as a river, that’s a huge river—and it’s very well placed, right where you need it, in the cities. If it can be recycled and put to use, the more direct the better.”
